# Comparison of the first Iranian native *Ornithobacterium rhinotracheale* vaccine with conventional vaccine: A challenge study

**DOI:** 10.14202/vetworld.2020.655-660

**Published:** 2020-04-12

**Authors:** N. Ghasemipour, H. Goudarzi, M. Banani, K. Asasi

**Affiliations:** 1 Department of Clinical Sciences, Poultry Diseases Research Center, Veterinary School of Shiraz University, Shiraz, Iran; 2 Department of Avian Diseases Research and Diagnosis, Razi Vaccine and Serum Research Institute, Agricultural Research, Education and Extension Organization, Karaj, Iran

**Keywords:** challenge, chickens, efficacy, *Ornithobacterium rhinotracheale*, polymerase chain reaction, vaccine

## Abstract

**Background and Aim:**

The best strategy to prevent or control an *Ornithobacterium rhinotracheale* (ORT) infection is vaccination. The present study aimed to compare the efficacy of the first Iranian inactivated ORT vaccine (Razi, Iran), which had been prepared from a native strain, with the Nobilis ORT Inac (Intervet, The Netherlands) through a challenge trial.

**Materials and Methods:**

Seventy-two 1-day-old specific pathogen-free White Leghorn chickens were used in this study. The birds were divided randomly into four groups. Following the vaccination and challenge of the birds, the efficacy of the Razi and the Intervet ORT vaccines was evaluated by serological, bacteriological, and molecular methods.

**Results:**

The antibody titer in vaccinated groups was determined to be significantly higher than unvaccinated birds. In addition, the difference in postmortem lesion scores between the vaccinated and unvaccinated birds was significant. The differences in the means of the antibody titers and postmortem lesion scores in birds that were vaccinated by the Razi and Intervet ORT vaccines were not significant.

**Conclusion:**

Considering the results of this study, it can be concluded that the Iranian native ORT vaccine was comparable to the Intervet vaccine. The Razi ORT vaccine has effectively decreased the duration of the ORT infection and can effectively protect the chickens against an ORT infection.

## Introduction

Respiratory tract diseases are commonly encountered health problems in poultry operations. *Ornithobacterium rhinotracheale* (ORT) is a Gram-negative pathogen microorganism that causes respiratory tract diseases in chickens and is associated with retarded growth, decreased egg production, increased mortality, and, inevitably, economic losses in poultry [1,2].

Unfortunately, most ORT strains are now resistant to most types of antibiotics [3,4]. Treatment of an ORT infection is really difficult and cannot be achieved effectively through antibiotic use. Probably, the best way to control ORT infections is by vaccination [5,6]. Although vaccination with an inactivated oil adjuvant vaccine was successful in reducing outbreaks of ORT [7], the major challenge in vaccine development against bacterial infections is the existence of different serotypes within a pathogen species[8]. The most challenging studies have concluded that experimental inoculation with ORT alone causes minimal pathogenic lesions in chickens. The severity of ORT lesions is enhanced when there is concurrent infection with respiratory viruses [9-11], so to more accurately evaluate the Razi vaccine; we designed the experimental challenge system with native ORT bacteria and the LaSota Newcastle disease virus (NDV).

The aim of our study was to compare the efficacy of the first Iranian native ORT vaccine with the conventional ORT vaccine through the challenge system.

## Materials and Methods

### Ethical approval

The samples were collected under the usual veterinary service work in Iran; no specific permission required for such type of study. The present work was performed according to the national standards of Iran.

### Chickens

Seventy-two 1-day-old specific pathogen-free (SPF) White Leghorn chickens (Venky’s Company, India) were used in this study. The birds were divided randomly into four groups (18 chicks per group). The birds were housed at the Poultry Research Unit of Razi Vaccine and Serum Research Institute in separate specific cages in isolation rooms. The chickens had free access to sterile water and disinfected food and were not administered any other vaccination or treatment during the experiment. The water was sterilized using an autoclave, and the food was disinfected in special containers using formaldehyde.

### Bacteria

The isolate ORT R87-7/1387(JF 810491) was used. This strain (SerotypeA) was stored at ‒70°C. The organism was retrieved from frozen suspension and cultured for 48h at 37°C on Columbia agar (Oxoid Ltd., Basingstoke, UK), with 5% sheep blood, in 5% CO_2_ atmosphere. The colonies were then transferred into the brain heart infusion (Oxoid Ltd., Basingstoke, UK) for 24h, at 37°C, with agitation. At that point, the bacterial challenge inoculums were prepared by washing cultured bacteria twice in phosphate-buffered saline, and a suspension containing 1×10^10^ colony-forming units (CFU) per 0.5ml was prepared.

### Experimental design

At the age of 14days old, the chickens of GroupV1 were vaccinated by subcutaneous injection in the neck region with 0.3ml (containing approximately 1×10^10^cells) of inactivated oil adjuvant of the native ORT vaccine. That vaccine was produced with SerotypeA of the ORT that was isolated during past respiratory disease outbreaks on poultry farms in various provinces of Iran. The birds of GroupV2 were injected by the same method with 0.25ml (containing approximately 1×10^10^cells) of the Nobilis ORT inactivated vaccine (Intervet International B. V., Boxmeer, The Netherlands) containing inactivated ORT SerotypeA bacteria in a mineral oil adjuvant. The birds of GroupsC_1_ and C_2_ were injected by the same method with 0.3ml of the sterile physiological saline. At 42days of age (at 4weeks after vaccination), the birds of GroupV_1_, V_2_, and C_1_ were challenged with a 10^6^ EID_50_ per dose of the LaSota NDV vaccine by the ocular route and 1×10^10^ CFU/0.5ml of the ORT by the intratracheal route. Each bird in GroupC_2_ was given one drop of sterile, distilled water by the intraocular route and 0.5ml of sterile, physiological saline by the intratracheal route.

### Sampling

After the challenge, typical clinical signs affected chickens, including respiratory signs (rale and gasping), and ruffled feathers were observed. Blood samples were collected from the brachial vein of birds at 2weeks of age before vaccination, at 6weeks of age (before the challenge), and at days 2, 4, 6, 8, 10, and 12 after challenge (AC). The serum samples were tested to evaluate the antibody titer against *Ornithobacteriosis*, using the commercial ELISA system. In addition, three chickens in each group were randomly killed at 2, 4, 6, 8, 10, and 12days AC (DAC) by cervical dislocation. After a postmortem investigation of the chickens, the samples of the trachea, lungs, air sacs, liver, and spleen of those birds were aseptically collected immediately and examined by bacteriological and molecular methods.

### Postmortem investigation of symptoms

Postmortem investigations were performed at 2, 4, 6, 8, 10, and 12 DAC, and the lesions were scored as described by van Empel *et al*. [12]. Airsacculitis was scored for the thoracic and abdominal air sacs separately. The average lesion scores were given as a percentage of the maximum possible score.

### Bacteriological analysis

For microbiological analysis, the samples of the lungs, trachea, air sacs, liver, and spleen were aseptically inoculated on blood agar supplemented with 5% sheep blood, 5µg/ml of gentamicin, and 5µg/ml of polymyxin B. The plates were incubated in 10% CO_2_ atmosphere at 37°C for at least 48h. Then, the suspicious colonies were subcultured, and the identities confirmed biochemically as previously described by van Empel *et al*. [13].

### Antibody detection

Serum samples were tested for the presence of the ORT antibody using the BioChek ORT Antibody Test Kit (The Netherlands) by following the manufacturer’s instructions.

### DNA extraction

The suspension of tissue samples and the ORT cultured broth was used for DNA extraction; 0.5ml of the suspension of tissue samples or the ORT cultured broth was transferred into Eppendorf tubes. The tubes were centrifuged at 13,000rpm for 15min; then, the sediments were transferred into new Eppendorf tubes and 100 µl of lysis buffer (20 mM Tris-pH8.0+150 mM NaCl+10 mM EDTA+0.2% SDS) were added to each tube. Following a 4-h incubation at 56°C, an equal volume of saturated phenol was added to the tubes and then centrifuged at 13,000rpm for 15min. The upper phase was transferred into a new Eppendorf tube. An equal volume of phenol and chloroform was added to the tube and then centrifuged at 13,000rpm for 15min. The supernatant was separated carefully, and then, an equal volume of phenol was added and centrifuged at 13,000rpm for 15min. The upper phase was transferred into a new tube. Subsequently, genomic DNA was precipitated with absolute ethanol and 0.3 M sodium acetate at ‒20°C for 20min. The mixture was then centrifuged at 13,000rpm for 10min, and the upper phase was discarded. The pellet was washed twice with 250 µl 0f 90% and 70% ethanol, respectively, and each step was followed by 5min of centrifugation. The pellet was dried and resuspended in 50 µl sterile, distilled water and used as a target DNA in polymerase chain reaction (PCR).

### Primers

The primers used in this study were designed by van Empel *et al*. [13]. The sequence of the primer pairs was as follows: OR 16S-F_1_ (5´-GAGAATTAATTTACGGATTAAG-3´) and OR 16S-R_1_ (5´-TTCGCTTGGTCTCCGAAGAT-3´). These primers amplify a 784bp fragment on the 16S rRNA gene of ORT.

### PCR

The PCR was performed in a master cycler gradient thermocycler (Eppendorf, Hamburg, Germany) in a total reaction volume of 25 µl containing 1 µl of DNA template sample, 1 µl of each primer (10 pmol), 1 µl deoxynucleotide triphosphates mix (10 mM), 1 µl MgCl_2_ (25 mM), 2.50 µl PCR buffer (10X), and 0.25 µl Taq DNA polymerase (1.25 units). All reagents were purchased from SinaClon Bioscience Co., Tehran, Iran. Amplification was obtained with an initial denaturation step at 94°C for 5min, followed by 30cycles at 94°C for 1min (denaturation), 54°C for 1min (annealing), and 72°C for 2min. The final extension cycle was at 72°C for 10min. Then, 10 µl of PCR products were separated by electrophoresis (100 volts for 1h) in 1% agarose gel (CinnaGen Co., Tehran, Iran) stained with 0.50µg/ml safe stain. DNA fragments were visualized by Ultraviolet Transillumination (UVitec, Cambridge, UK) and compared with a 100bp DNA ladder. The isolate ORT R87-31/1387(JF 810491) and distilled water were used as the positive and negative controls.

### Statistical analysis

The results were analyzed statistically using the SPSS software (version22.0; SPSS Inc., Chicago, USA). The comparison of the means of ORT ELISA titers between and within groups was performed through ANOVA analysis, and the Duncan method was used for the cooperation of mean ORT ELISA titers of groupsAC. Postmortem lesion scores in experimental groups were analyzed using a Chi-square analysis. In all tests, results with p<0.05 were regarded as statistically significant.

## Results

### Clinical findings

None of the chickens died during the experiment. However, respiratory signs, including rale and gasping, were not detected at 2, 4, 6, 8, 10, and 12 DAC in chickens in the vaccinated groups. Clinical signs (rale, gasping, and ruffled feathers) were seen in birds of the positive control group, starting from 2 DAC, gradually increasing until 8 DAC, and lasting for 12 DAC. In birds in the vaccinated groups, the ruffled feathers were detected at 2 and 4 DAC, then this declined and was absent at 6 DAC. None of the clinical findings were detected in birds of the negative control group in DAC.

### Postmortem examination

The results of postmortem lesion scores and the percentage of the maximum possible score of thoracic airsacculitis, abdominal airsacculitis, and pneumonia are presented in Table-1. No macroscopic lesions were observed in air sacs and lungs in the negative control group, which was not vaccinated with any of the vaccines and was not challenged with ORT, whereas the lesion scores were at a maximum in the challenge control group. The lesion scores on airsacculitis and pneumonia were at a minimum in the vaccinated groups. The comparison of the difference in lesion scores was between vaccinated groups (V_1_ vs. V_2_), so between the vaccinated groups with the negative control group (V_1_ and V_2_ vs. C_2_) were not significant (Table-2).

**Table-1:** Postmortem lesion scores in the experimental groups after challenge with the ORT.

Groups	Thoracic air sacs					Abdominal air sacs					Lungs				
		
	NO	Score 0	Score 1	Score 2	% MPS	NO	Score 0	Score 1	Score 2	% MPS	NO	Score 0	Score 1	Score 2	% MPS
V_1_	18	15	3	0	8.3	18	16	2	0	2.8	18	16	2	0	5.6
V_2_	18	16	2	0	5.6	18	18	0	0	0	18	17	1	0	2.8
C_1_	18	3	2	13	77.8	18	4	1	13	75	18	8	3	7	42.7
C_2_	18	18	0	0	0	18	18	0	0	0	18	18	0	0	0

MPS=Maximum possible score, NO=Number of birds, V_1_=Razi ORT vaccine, V_2_=Intervet ORT vaccine, C_1_=Positive control, C_2_=Negative control

**Table-2:** Comparison of the postmortem lesions between experimental groups after challenge with the ORT.

Comparison of groups	Postmortem lesions		

	Thoracic air sacs	Abdominal air sacs	Lungs
V_1_ versus V_2_	0.232^ns^	1.029^ns^	0.364^ns^
V_1_ versus C_1_	21.200**	21.048**	9.967**
V_1_ versus C_2_	3.273^ns^	1.029^ns^	2.118^ns^
V_2_ versus C_1_	21.895**	22.909**	11.240**
V_2_ versus C_2_	2.118^ns^	0.000^ns^	1.029^ns^
C_1_ versus C_2_	25.714**	22.909**	13.846**

ns=Not significant,

** =Significant p<0.01. V_1_=Razi ORT vaccine, V_2_=Intervet ORT vaccine, C_1_=Positive control, C_2_=Negative control

### Serology

The antibody titer in different phases of the study is presented in Table-3. Before vaccination, the antibody to ORT was negative in all of the experimental groups. The vaccinated birds had a higher antibody titer when compared with unvaccinated groups and were significantly different, but the difference between vaccinated groups was not significant (Table-4). The mean antibody titer in vaccinated groups rose after the challenge with ORT, but no significant difference was found within vaccinated groups before and after the challenge (Table-5).

**Table-3:** Mean ORT ELISA titer in different phases of study.

Group	Phases		

	BV	BC	AC
V_1_	0.00±0.00	12416.6±1971.6	14184.6±1567.81
V_2_	0.00±0.00	14660.5±1239.4	16079.5±1175.83
C_1_	0.00±0.00	0.00±0.00	9481.60±449.89
C_2_	0.00±0.00	0.00±0.00	0.00±0.00

BV=Before vaccination, BC=Before challenge, AC=After challenge, V_1_=Razi ORT vaccine, V_2_=Intervet ORT vaccine, C_1_=Positive control, C_2_=Negative control

**Table-4:** Variance analysis for ORT ELISA titer after challenge (AC).

S.O.V.	df	Mean square	F	Sig.
Between groups	3	33283.844	185.749	0.000
Within groups	36	179.187		
CV%		15.68%		

Sig.=Significance

**Table-5:** Comparison of mean ORT ELISA titer within the vaccinated groups before and after challenge with the ORT.

Group	t-value	df	Significance
V_1_	‒0.702^ns^	18	0.492
V_2_	‒0.831^ns^	18	0.417

ns=Non-significance, V_1_=Razi ORT vaccine, V_2_=Intervet ORT vaccine

### Bacteriology

The results of bacteriological isolation are displayed in Table-6. ORT was never isolated from the spleen and liver of the chickens at 2, 4, 6, 8, 10, and 12 DAC in all of the experimental groups. Like in the vaccinated groups, ORT was not recovered from the lungs, trachea, and air sacs of the birds. In the positive control group, ORT was isolated at 2 and 4 DAC from the trachea and air sacs of the birds. ORT was only found at 4 DAC from the lungs of the chickens in the positive control group. In the negative control group, ORT was not found in DAC.

**Table-6:** Results of the culture of the samples after challenge with the ORT.

Organs	Days (AC) 2				Days (AC) 4				Days (AC) 6				Days (AC) 8				Days (AC) 10				Days (AC) 12			
					
	V_1_	V_2_	C_1_	C_2_	V_1_	V_2_	C_1_	C_2_	V_1_	V_2_	C_1_	C_2_	V_1_	V_2_	C_1_	C_2_	V_1_	V_2_	C_1_	C_2_	V_1_	V_2_	C_1_	C_2_
Trachea	‒	‒	+	‒	‒	‒	+	‒	‒	‒	‒	‒	‒	‒	‒	‒	‒	‒	‒	‒	‒	‒	‒	‒
Lungs	‒	‒	‒	‒	‒	‒	+	‒	‒	‒	‒	‒	‒	‒	‒	‒	‒	‒	‒	‒	‒	‒	‒	‒
Airsacs	‒	‒	+	‒	‒	‒	+	‒	‒	‒	‒	‒	‒	‒	‒	‒	‒	‒	‒	‒	‒	‒	‒	‒
Liver	‒	‒	‒	‒	‒	‒	‒	‒	‒	‒	‒	‒	‒	‒	‒	‒	‒	‒	‒	‒	‒	‒	‒	‒
Spleen	‒	‒	‒	‒	‒	‒	‒	‒	‒	‒	‒	‒	‒	‒	‒	‒	‒	‒	‒	‒	‒	‒	‒	‒

AC=After challenge, V_1_=Razi ORT vaccine, V_2_=Intervet ORT vaccine, C_1_=Positive control, C_2_=Negative control

### Molecular analysis

After the challenge, the molecular analysis (PCR) was performed on different parts of the respiratory system (lungs, trachea, and air sacs) and visceral organs (spleen and liver) of the chickens. Finally, ORT was only detected from samples of the lungs, trachea, and air sacs of birds at 2 DAC in the vaccinated groups. In the positive control group, ORT was detected from the lungs, trachea, and air sacs at 2 and 4 DAC. In the negative control group, ORT was not detected in DAC (Table-7).

**Table-7:** Results of the PCR of the samples after challenge with the ORT.

Organs	Days (AC) 2				Days (AC) 4				Days (AC) 6				Days (AC) 8				Days (AC) 10				Days (AC) 12			
					
	V_1_	V_2_	C_1_	C_2_	V_1_	V_2_	C_1_	C_2_	V_1_	V_2_	C_1_	C_2_	V_1_	V_2_	C_1_	C_2_	V_1_	V_2_	C_1_	C_2_	V_1_	V_2_	C_1_	C_2_
Trachea	+	+	+	‒	‒	‒	+	‒	‒	‒	‒	‒	‒	‒	‒	‒	‒	‒	‒	‒	‒	‒	‒	‒
Lungs	+	+	+	‒	‒	‒	+	‒	‒	‒	‒	‒	‒	‒	‒	‒	‒	‒	‒	‒	‒	‒	‒	‒
Airsacs	+	+	+	‒	‒	‒	+	‒	‒	‒	‒	‒	‒	‒	‒	‒	‒	‒	‒	‒	‒	‒	‒	‒
Liver	‒	‒	‒	‒	‒	‒	‒	‒	‒	‒	‒	‒	‒	‒	‒	‒	‒	‒	‒	‒	‒	‒	‒	‒
Spleen	‒	‒	‒	‒	‒	‒	‒	‒	‒	‒	‒	‒	‒	‒	‒	‒	‒	‒	‒	‒	‒	‒	‒	‒

AC=After challenge, V_1_=Razi ORT vaccine, V_2_=Intervet ORT vaccine, C_1_=Positive control, C_2_=Negative control

## Discussion

An ORT infection is considered of the emerging diseases of poultry. Various studies on the control of an ORT infection in poultry through vaccination have been described [7,8]. The vaccination of chickens was effective and protected from pathologic changes [1]. The major challenge in vaccine development against bacterial infections is the existence of different serotypes within a pathogen species [8]. Since SerotypeA of ORT is the most prevalent serotype in chickens [1], this serotype chooses to produce the inactivated vaccine. The Razi ORT vaccine had been prepared from the native strain. This native strain (SerotypeA) was isolated during past respiratory disease outbreaks on poultry farms in various provinces of Iran.

In the present challenge trial, no mortality was observed in chickens. The severity of clinical signs, the duration of the disease, and the mortality of ORT outbreaks are extremely variable depending on the strains of bacteria and the breed of the chickens [1]. The clinical signs of birds in this study are similar to, but generally milder than, those seen in filed cases. This difference may be attributed to the often inadequate environmental and management conditions (high animal density, inadequate ventilation, and high ammonia levels) and additional pathogens encountered in the field, exacerbating any disease that has been brought about. The birds used in this study were SPF and kept in spacious rooms with high-efficiency particulate air-filtered air with no extraneous pathogens interplaying.

Based on the results of the postmortem examination, the airsacculitis and pneumonia were most severe in chickens of the positive control group, whereas the airsacculitis and pneumonia were at aminimum in the experimental groups vaccinated with the Razi andthe Intervet vaccines. Results of this study show that in vaccinated groups, gross lesions occurred less frequently than in unvaccinated birds, and vaccinating chickens had a striking effect on airsacculitis and pneumonia, in agreement with the findings of Murthy *et al*.[14] and Sprenger *etal*.[15]. Significant differences in lesion scores were seen between the vaccinated groups and the unvaccinated birds that were challenged with ORT and ND. Asignificant difference in lesion scores was not seen between the birds that were vaccinated with the Razi ORT vaccine and the birds that were vaccinated with the Intervet vaccine (Table-2). After the challenge in the vaccinated groups, a significant reduction in pathology was observed, although the organ lesions were not reduced to zero. Our results were in agreement with the study of Hegazy *et al*. [16].

**Figure 1:**
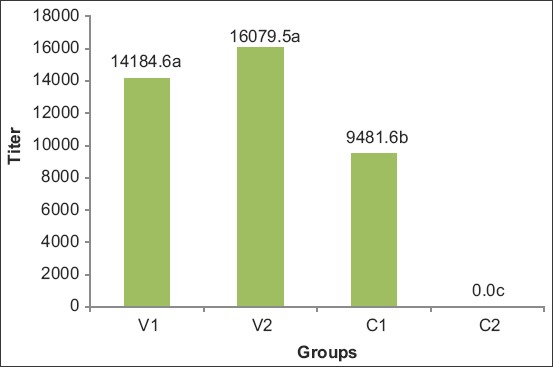
Mean comparison of ORT ELISA titer in experimental groups after challenge with the ORT V_1_, Razi ORT vaccine; V_2_, Intervet ORT vaccine; C_1_, positive control; C_2_, negative control

The titer of antibody to ORT in the blood sera of vaccinated birds was determined to be significantly higher than that of unvaccinated chickens(Figure-1). In the present study, before vaccination, antibodies to ORT were negative in all of the experimental groups, but the chickens that received vaccines had a higher antibody titer when compared with the negative control group and were significantly different. Our findings of the effective role of the vaccine to evoke high antibody titers against ORT were correlated with the reports of Cauwerts *et al*.[6], Schuijffel *et al*. [5], and Erganis *et al*. [7]. After vaccination and after the challenge, no significant difference was observed between the titer of antibody to the ORT in the blood sera of vaccinated groups.

After the challenge, bacteriological (culture) and molecular (PCR) analyses were performed on the samples of the respiratory organs (lungs, air sacs, and trachea) and visceral organs (liver and spleen). In all of the experimental groups, ORT was not detected by the culture and PCR methods from the visceral organ samples taken in the DAC. This is in agreement with the study of Hegazy *et al*. [16] and Umali *et al*. [4]. The bacterium primarily infects the trachea, lungs, and air sacs, but can also manifest as a systemic disease [8,12]. The ORT bacterium is most commonly isolated from the trachea and lungs of naturally or experimentally infected birds [17-19]. In our study, the bacterium was not isolated from the respiratory system by the culture in the vaccinated groups in the DAC. In vaccinated groups, ORT was detected by the PCR at 2DAC, but in the positive control group, the ORT was isolated by the culture and PCR from the respiratory organs until 4DAC. Like in the vaccinated groups, the bacterium was only detected in PCR from samples of the lungs, trachea, and air sacs at 2DAC. ORT can normally isolate only at an early stage of the infection and attempts to recover it at a later stage often fail[1]. The use of PCR enables the attainment of positive results in samples from the later days after infection, which remained negative in bacteriological analyses. The PCR technique additionally detected the bacterial genetic material in respiratory organs at 4DAC in unvaccinated groups. The OR16S-F1 and OR16S-R1 primers combination used in our study were very specific in amplifying a 784bp fragment of the 16S rRNA gene of ORT [20].

## Conclusion

Considering the results of the clinical signs, postmortem lesions, serology, culture, and PCR in this study, it can be concluded that no significant difference was found been between the Razi ORT vaccine and the Intervet ORT vaccine. The vaccination of the birds by the Iranian native ORT vaccine has effectively decreased the duration of the ORT infection and can effectively protect the chickens against an ORT infection.

## Authors’ Contributions

KA, MB, and HG designed and performed the study. MB and HG participated in challenging, collecting data. KA, MB, and HG participated in the interpretation of data. NG collected samples, participated in bacteria isolation, performed PCR, analysis of data, and draft of the manuscript. KA wrote and revised the manuscript. All authors read and approved the final manuscript.
